# Effect of a Fibroin Enzymatic Hydrolysate on Memory Improvement: A Placebo-Controlled, Double-Blind Study

**DOI:** 10.3390/nu10020233

**Published:** 2018-02-17

**Authors:** Yong Koo Kang, Boo Yong Lee, Luke R. Bucci, Sidney J. Stohs

**Affiliations:** 1 BrainOn Inc., Ltd., 403 Isbiz Tower, 23 Seonyuro49-gil, Youngdeungpo-gu, Seoul 07206, Korea; yongkooo@paran.com (Y.K.K.); byl812@hanmail.net (B.Y.L.); 2 Inner Path Nutrition, Reno, NV 89523, USA; lukebucci@earthlink.net; 3 School of Pharmacy and Health Professions, Creighton University Medical Center, Omaha, NE 68178, USA

**Keywords:** auditory verbal learning, *Bombyx mori* (silkworm), complex figure test, dose response, fibroin enzymatic hydrolysate, drawing/recall, learning gradient, memory quotient, memory retention, retrieval efficiency

## Abstract

The consumption of a specifically prepared silk fibroin protein enzymatic hydrolysate (FPEH) has been reported to improve cognitive function in healthy humans. The objective of the current study is to evaluate the dose-dependent effects of the FPEH on memory. Healthy adults with an average age of approximately 55 years were administered doses of 0, 280, 400 and 600 mg of FPEH per day in two divided doses for 3 weeks. The Rey–Kim Auditory Verbal Learning Test and the Rey–Kim Complex Figure Test of the Rey–Kim Memory Test were used to evaluate memory at baseline and after 3 weeks. The scores for each test were combined into the memory quotient score (MQ). Learning gradient, memory maintenance, retrieval efficacy, and drawing/recall scores were also compared. After 3 weeks of FPEH, dose-dependent increases were observed for the MQ, the learning gradient, the numbers of words remembered, the retrieval efficiency, and drawing/recall. The optimal dose for FPEH was 400 or 600 mg, depending on the end point measured. No adverse effects were reported. FPEH significantly improved measurements of memory in healthy adults by 3 weeks at doses over 280 mg daily, with an apparent plateau effect at 400–600 mg daily.

## 1. Introduction

Historically, silk and silk protein hydrolysates have been consumed as food and as traditional Asian medicine for health benefits, including in use as tissue protectants and anticancer agents and to enhance the immune system [1,2]. Silkworm cocoons of the silkworm moth, *Bombyx mori*, are composed almost entirely of protein with the inner core of the silk strand consisting primarily of the protein fibroin [3,4]. Fibroin proteins are interconnected into a continuous, polymeric mesh network that forms a cocoon from a single silk strand with a length of up to 1000 m [3,4]. Silkworm fibroin has a very specific sequence of repeated blocks of hydrophobic polyglycine (24–35 residues) and polyalanine (8–10 residues), primary sequences that form a common protein structure called beta-sheets. The sheets are arranged to be adjacent when folded. Other amino acids including l-serine and l-tyrosine occupy key positions in the protein strand sequence and account for the folding of fibroin into continuous, long strands of silk protein. These four amino acids comprise over 90% of the weight of fibroin protein.

In recent years, it has been reported that a specifically prepared, proprietary silk fibroin protein enzymatic hydrolysate (FPEH) improves cognitive function in normal, healthy humans [5,6,7,8,9,10]. The enzymatic hydrolysis results in short strands of the protein with a molecular weight range of 500–5000 daltons. These studies included children, high school/college students, adults and seniors ranging in age from about 9 to 72 years. The study designs were randomized and placebo controlled with acute and 3–16 week durations. 

Eight separate, controlled human studies have found that FPEH at doses of 200–400 mg daily administered acutely or for 3–16 weeks significantly improved mental function from baseline and placebo groups by validated measurements [5,6,7,8,9,10]. Furthermore, an animal study showed improvements or restoration of various memory deficits by the oral administration of FPEH [11]. FPEH was well tolerated, with no differences in adverse effects as compared to subjects administered the placebo.

The purpose of the current study is to evaluate the dose-dependent effectiveness of the FPEH for memory improvement in healthy adults, using a validated memory test. Doses of 280, 400 and 600 mg/day were given, with the 600 mg dose being higher than doses used in previous studies [5,6,7,8,9,10]. The study assessed memory effects over a wide age range under identical experimental conditions and was designed to validate results of previous studies.

## 2. Materials and Methods

### 2.1. Study Material

The FPEH was provided by BrainOn Co., Ltd., 403 Isbiz Tower, 23 Seonyuro49-gil, Youngdeungpo-gu, Seoul 07206, Korea. The product, also known as BF-7, was prepared on the basis of the procedure of Yeo et al. [12], and it is sold under the trade name of Cera-Q (Novel Ingredients LLC, East Hanover, NJ, USA). The study material has been approved by the Korean Ministry of Food and Drug Safety as a food ingredient with no limits on the intake amount.

### 2.2. Study Subjects

The protocol for this study was approved (12 January 2017) by the Korean Ministry of Health and Welfare Public Institutional Review Board (Registration No. 2016-0458-010). Healthy adult volunteers between the ages of 28 and 92 years were recruited for the study by visiting religious meetings, friendly gatherings and senior citizen centers, providing a wide range of individuals within the general population. The intent was to determine the effectiveness of the FPEH over a wide age range. Age-related differences in memory retrieval efficiency were accounted for by using an age conversion table. Exclusionary criteria included current or past history of neurological disorders or traumatic brain injury; history of epileptic events, seizures or sleep disorders; history of blood clotting disorders; being pregnant or lactating; use of cholesterol lowering drugs; use of anti-hypertensive drugs; allergy to silk; use of anti-inflammatory drugs on an on-going basis; and consumption of herbal or medicinal products related to memory. The protocol was explained in detail to all subjects, and written informed consent was obtained. Before beginning the study, baseline assessments of general health, vital signs, and weight were determined.

### 2.3. Treatment

A total of 76 subjects was recruited for the study and were randomized into 4 groups of 19 individuals. The study was placebo controlled, randomized and double-blind with the subjects receiving identically appearing capsules. The placebo consisted of dextrin. The groups of subjects orally consumed either the placebo, or 280, 400 or 600 mg of the purified, specifically prepared, proprietary FPEH daily, in two divided doses, morning and evening without regard to meals, for 3 weeks (21 days). Three weeks of treatment was selected on the basis of previous studies that have demonstrated significant, beneficial effects as early as 3 weeks [6,9,10]. All subjects were assessed mid-way through the study to evaluate the level of compliance. A total of 15 subjects were dropped from the study because of non-compliance (taking less than 70% of the allocated study material): 5 from the placebo group, 3 subjects each from the 280 and 600 mg FPEH groups, and 2 subjects from the 400 mg FPEH group. Compliance was assessed on the basis of capsule counts in the containers of each subject at the mid-point and the end of the 3 weeks and by oral discussions with the subjects. 

### 2.4. Assessment

All subjects completed the Rey–Kim Memory Test [13,14] at baseline and after completion of 3 weeks of consumption of their treatment. This test is the validated Korean version of the Rey Memory Test. This test was used because it provides a measure of memory for content, location and sequence, utilizing both verbal as well as non-verbal memory performance. With this test, verbal memory performance is assessed with the Korean version of the Rey Auditory Verbal Learning Test (KAVLT), while non-verbal memory performance is evaluated with the Korean version of the Rey Complex Figure Test (KCFT) [13,14]. The scores for each test are combined and converted into the memory quotient (MQ), which is an index of complex memory functioning involving the recognition of both abstract and meaningful material and short-term recall. 

The KAVLT was composed of repeated trials for a total of five tests per time point, a delayed remembrance test, and a delayed recognition test. The KCFT test was composed of copying a complex figure and then drawing the figure from memory immediately and 20 min later for an immediate and delayed test of visuospatial abilities, memory, attention, planning and working memory (executive functions). The standard 36-point scoring system was used for the KCFT tests [15]. 

The verbal and figure aspects of memory were each tested, and each subsection score was calculated, converted into a composite score depending on each study subject’s age, and displayed as the MQ. The MQ serves as the most direct index of memory and concentration. The learning gradient measures how quickly the examinee remembers from repetition. The learning gradient is expressed as the percentage of the difference in remembered words from Trial 1 and Trial 5 of the KAVLT test before and after 3 weeks. Memory retention measures the ability to maintain what was put into memory by determining the difference in the number of words remembered after the intake of FPEH for 3 weeks as compared to before the intake (time zero). Retrieval efficiency measures the ability to accurately retrieve and use what was input into memory. Retrieval efficiency is calculated using the KAVLT delayed recognition raw score minus the delayed remembrance raw score, followed by conversion using the age conversion table. A higher percentage of accuracy indicates a greater ability to accurately retrieve the memory needed. Drawing by copying and two recalls measures the correlation between the ability to understand figures and memory. In the KCFT, a composite score of the percentage of correct responses from three separate drawings of the complex figure—copying, immediate recall and delayed recall—represents short-term memory.

### 2.5. Statistical Analysis

All data are expressed as means ± standard error of the means (SEM). A generalized linear model (PROC-GLM, ANOVA) from the Statistical Analysis System (SAS, version 9.3, SAS Institute Inc., Cary, NC, USA) was used to analyze the data. The number of subjects that completed the study in each group was sufficient for statistical processing based on a post hoc power analysis. When statistical differences (*p* < 0.05) determined by ANOVA using Tukey’s post hoc test were found, comparisons among groups were subsequently conducted using the least-squares or Duncan methods; *ω*^2^ effect size calculations were calculated from SAS ANOVA tables as per Albers and Lakens [16].

## 3. Results

### 3.1. Subject Demographics

Fourteen subjects (3 males and 11 females) completed the study in the placebo group, while 16 subjects completed the study in the 280 mg (3 males and 13 females) and 600 mg (4 males and 12 females) FPEH groups, and 17 subjects (5 males and 12 females) completed the study in the 400 mg FPEH group. The average age in the placebo group was 59.7 ± 16.7 years, while the average ages in the 280, 400 and 600 mg FPEH groups were 55.1 ± 9.1, 51.5 ± 11.6, and 54.7 ± 13.3 years, respectively. Overall, the average age was 55.2 ± 12.6 years, ranging from 28 to 92 years, with females comprising 76.1% of subjects.

### 3.2. Effects of FPEH on Memory Quotient of Rey–Kim Memory Test

**Figure 1:**
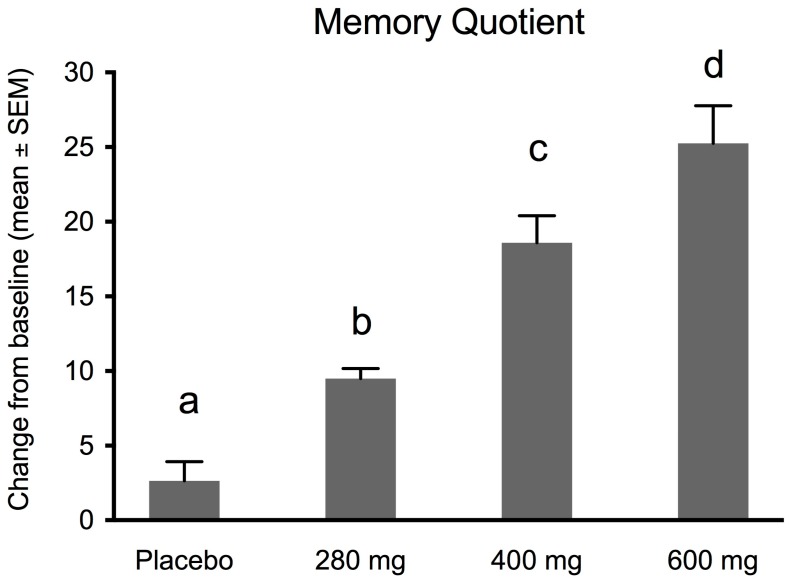
Changes in memory quotient (MQ) before and after silk fibroin protein enzymatic hydrolysate (FPEH) intake at daily doses of 0, 280, 400 and 600 mg for 3 weeks. Each value is the mean with the standard error of the mean (SEM). Groups with a different alphabetical letter are statistically significantly different on the basis of group tests following ANOVA (*F* = 32.97; *df*_total_ = 62; *p* < 0.00013). Between group *p* values were the following: placebo: 280 mg, *p* < 0.00015; placebo: 400 mg, *p* < 0.0001; placebo: 600 mg, *p* < 0.0001; 280 mg: 400 mg, *p* < 0.0001; 280 mg: 600 mg, *p* < 0.0001; 480 mg: 600 mg, *p* < 0.0378.

The effect of FPEH as a function of dose on the MQ is presented in Figure 1. As can be seen in Figure 1, significant dose-dependent changes in the MQ between baseline and the end of the study were observed for each FPEH group. The placebo group exhibited a non-significant 2.5% increase (*p* = 0.0599). The 280 mg FPEH group exhibited an 8.9% increase in the MQ (*p* < 0.0001). The 400 mg FPEH group exhibited a 17.5% increase in the MQ (*p* < 0.0001). A 24.8% increase in the MQ occurred for the 600 mg FPEH group (*p* < 0.0001). When the differences in the MQ before and after intake for subjects in each group were plotted (Figure 1), the dose-dependent increases were statistically significant from one another (ANOVA: *F* = 32.97; *df*_total_ = 62; *p* < 0.00013). The *ω*^2^ effect size was 0.605, a large value (>0.40) for ANOVA-derived effect sizes.

### 3.3. Effects of FPEH on Learning Gradient of Rey–Kim Memory Test (KAVLT)

**Figure 2:**
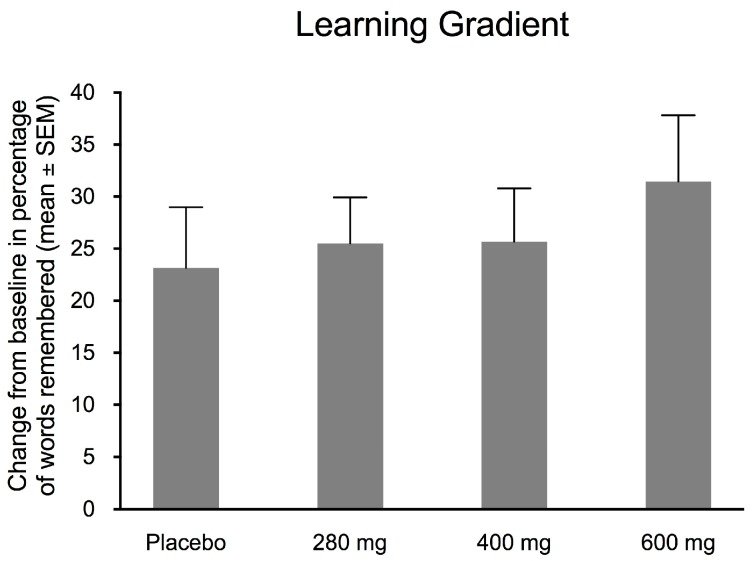
Learning gradient as the percentage of words remembered per trial after intake of daily doses of 0, 280, 400 and 600 mg of fibroin protein enzymatic hydrolysate (FPEH) for 3 weeks is exhibited on the *y*-axis. The data are presented as mean values with the standard error of the mean (SEM). On the basis of ANOVA (*F* = 2.8164; *df*_total_ = 62; *p* = 0.7440), the learning gradient changes with increasing dose were not statistically significant.

The Rey–Kim Memory Test carries out the KAVLT word, repeating trials five times at baseline and again after 3 weeks. As previously noted, the learning gradient is the percentage of the difference in remembered words from Trials 1 and 5 of the KAVLT test before and after 3 weeks. Figure 2 shows the learning gradient scores for each group. An increase in the learning gradient was observed for the 600 mg dose of FPEH as compared to the placebo and the two lower doses, but the difference was not significant by ANOVA (*F* = 0.413; *df*_total_ = 62; *p* = 0.7440). The *ω*^2^ ES (effect size) was −0.029: no effect size.

**Figure 3:**
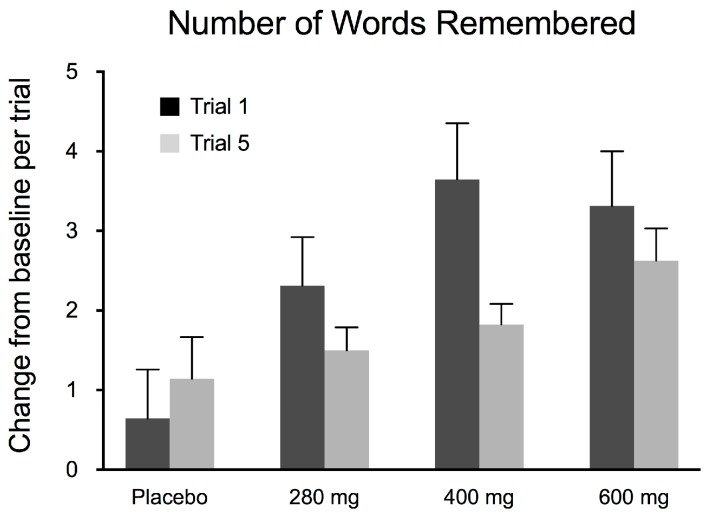
Changes in numbers of words remembered, before and after 3 weeks of fibroin protein enzymatic hydrolysate (FPEH), in Trials 1 (black bars) and 5 (gray bars) of the Korean version of the Rey Auditory Verbal Learning Test (KAVLT) is exhibited on the *y*-axis. The change in each group in the number of words remembered was assessed by ANOVA and was significant for Trials 1 (*p* = 0.0126) and 5 (*p* = 0.0463). Each value is the mean with the SEM.

Trials 1 (black bars) and 5 (gray bars) are compared to see how the number of words remembered differed before and after the 3 week intake of FPEH as another measure of the learning gradient (Figure 3). For Trial 1 (black bars), the results show that a dose-dependent increase in the number of words remembered occurred, reaching a maximum at the 400 mg daily dose of FPEH (ANOVA: *F* = 3.9300; *df*_total_ = 62; *p* = 0.0126). The *ω*^2^ effect size was 0.123, a small–medium value (0.10–0.25) for ANOVA-derived effect sizes. As compared to the placebo group, the 280 mg dose increase showed a non-significant trend compared to the placebo (*p* = 0.0655), but the 400 mg (*p* = 0.0040) and 600 mg (*p* = 0.0080) FPEH treatment groups exhibited statistically significant increases that did not differ significantly from each other (*p* = 0.7371).

Trial 5 (gray bars) of the Rey–Kim Memory Test showed that as the daily silk FPEH intake amount increased, the number of words remembered significantly increased (ANOVA: *F* = 2.8258; *df*_total_ = 62; *p* = 0.0463; Figure 3). The *ω*^2^ effect size was 0.081, below a small value (0.10) for ANOVA-derived effect sizes. The 600 mg daily treatment group exhibited the highest number of words remembered. The increase in the number of words remembered for Trial 5 was statistically significant as compared to the placebo control group only for the 600 mg FPEH treatment group (*p* = 0.0314). The difference between the 280 and 600 mg FPEH treatment groups was also statistically significant (*p* = 0.0316).

### 3.4. Effects of FPEH on Memory Retention of Rey–Kim Memory Test

**Figure 4:**
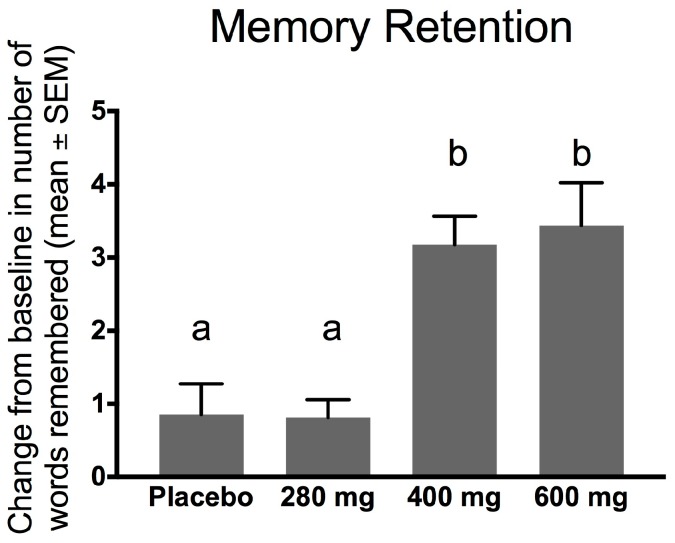
Memory retention measured as change in number of words remembered before and after 3 weeks of daily ingestion of fibroin protein enzymatic hydrolysate (FPEH) is exhibited on the *y*-axis. Significant differences between groups were assessed by ANOVA (*p* < 0.0001). Groups with different alphabetical letters are statistically significantly different (*p* < 0.05). The data are presented as means ± SEM.

Memory retention indicates how well the examinee maintained what was remembered from the Korean version of the Rey Auditory Verbal Learning Test (KAVLT) by assessing the difference in the number of words remembered before and after the intake of FPEH for 3 weeks (Figure 4). Significant differences between groups were assessed by ANOVA (*F* = 11.2503; *df*_total_ = 62; *p* < 0.0001). The *ω*^2^ effect size was 0.328, a medium–high value (0.25–0.40) for ANOVA-derived effect sizes. These results indicate that the 400 mg (*p* = 0.0003) and 600 mg (*p* = 0.0016) FPEH treatment group subjects remembered significantly more words than the placebo group subjects and were more able to maintain memory of words after 3 weeks of supplementation.

### 3.5. Effects of FPEH on Retrieval Efficiency of Rey–Kim Memory Test

**Figure 5:**
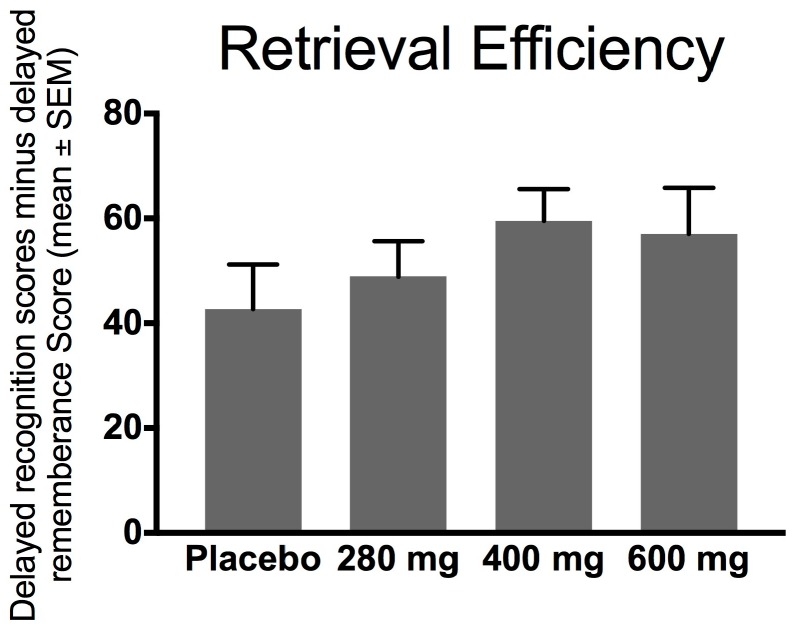
Retrieval efficiency calculated using the Korean version of the Rey Auditory Verbal Learning Test (KAVLT) delayed recognition raw score minus delayed remembrance raw score followed by conversion using the age conversion table is exhibited on the *y*-axis. The change in retrieval efficacy before and after intake of fibroin protein enzymatic hydrolysate (FPEH) for 3 weeks was not significant between groups by ANOVA (*p* = 0.3959). Each value is the mean with the SEM.

Retrieval efficiency measures how well the remembered information from the Rey Auditory Verbal Learning Test (RAVLT) could be applied to other information, with a higher percentage indicating a greater ability to accurately retrieve the memory needed. As compared to the placebo group, the FPEH treatment group subjects showed a dose-dependent improvement in retrieval efficiency of up to 400 mg, but the increases were not statistically significant (ANOVA: *F* = 1.0080; *df*_total_ = 62; *p* = 0.3959; Figure 5). The *ω*^2^ effect size was 0.0004, a negligible value for ANOVA-derived effect sizes.

### 3.6. Effects of FPEH on Visuospatial Abilities, Attention and Memory (Executive Functions)

**Figure 6:**
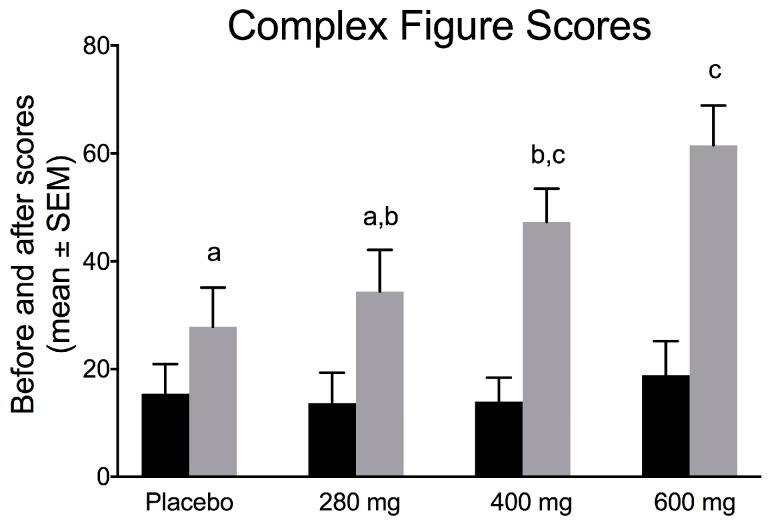
Korean version of the Rey Complex Figure Test (KCFT) memory test scores (as percentage of correct copying/recall) are exhibited on the *y*-axis. Baseline scores (black bars) for each group were similar. The percent change in drawing/recall score between groups before and after 3 weeks of daily fibroin protein enzymatic hydrolysate (FPEH) intake was increased in a dose-dependent manner (ANOVA = 0.0092). The 280 mg group change was not different from the placebo change (*p* = 0.5493); however, 400 mg (*p* = 0.0497) and 600 mg (*p* = 0.0032) group changes were significantly increased over the placebo group and were similar to each other (*p* = 0.1492). Each value is the mean with the SEM. Groups with a different alphabetical letter are statistically significantly different on the basis of *t*-tests.

The results for the KCFT, which represents short-term memory and integrated executive functions, are presented in Figure 6. Baseline scores for each group did not differ. A significant increase in complex figure scores was found by ANOVA analysis (*F* = 4.2073; *df*_total_ = 62; *p* =0.0092). The *ω*^2^ effect size was 0.132, a small–medium value (0.10–0.25) for ANOVA-derived effect sizes. The change in drawing/recall in the 280 mg FPEH treatment group, as compared to the placebo group, was not statistically significant (*p* = 0.5493). However, the 400 mg (*p* = 0.0497) and 600 mg (*p* = 0.0032) FPEH treatment groups exhibited statistically significant increases in KCFT scores from the placebo group. The 400 and 600 mg scores were not significantly different (*p* = 0.1492).

### 3.7. Side Effects

No adverse effects were reported by any of the subjects in the four study groups.

## 4. Discussion

The effects of the placebo and three doses (280, 400, and 600 mg) of silk FPEH for 3 weeks on memory and executive function in normal, healthy adults with an average age of about 55 years were determined by commonly used, validated tests. The results demonstrated that dose-dependent increases in several memory parameters were observed following the daily oral administration of FPEH. Significant dose-dependent increases were observed for the change from baseline of the MQ (Figure 1), the number of words remembered on the basis of Trial 1 (Figure 3), the increase in the number of words remembered over time (Figure 4), and the short-term memory on the basis of the ability to recall and draw a complex figure (Figure 6). Non-significant dose-dependent increases in memory were found for the other two measurements, namely, learning gradient (Figure 2) and retrieval efficiency (Figure 5).

The *ω*^2^ effect sizes were determined instead of typical *η*^2^ effect sizes, because the bias for false positive findings is less [16]. A large effect size was found for the difference in the MQ before and after 3 weeks, a medium–large effect size was found for memory retention, and small–medium effect sizes were found for the learning gradient Trial 1 and for changes in the complex figure scores. The other measurements showed a less-than-small effect size.

The greatest increases in the MQ, the learning gradient, the number of words remembered after Trial 5, and the ability to recall and draw a complex figure were observed after 3 weeks of daily doses of 600 mg of FPEH. A maximum effect was observed with a daily 400 mg dose of FPEH for numbers of words remembered after Trial 1, the increase in the number of words remembered, and the retrieval efficiency, with no significant difference between the 400 and 600 mg daily doses. The results indicate that a maximum beneficial effect of FPEH on memory can be achieved with a dose in the range of 400–600 mg in this study population.

This is the first published study to investigate a range of three doses for FPEH simultaneously. No previous study has examined a dose of 600 mg daily. At this dose, no adverse effects were observed or reported. Previous studies have used daily doses of 200 or 400 mg of FPEH. The current study confirmed the results of previous studies regarding the ability of FPEH to enhance memory and learning [5,6,7,8,9,10] This study extended the finding of efficacy for memory improvement from FPEH in a healthy population with ages as high as 92 years. Cognitive decline, specifically memory loss, is frequently associated with natural aging [17]. This study demonstrates the beneficial effects of FPEH over a wide age range as compared to previous studies that used more selective age groups [5,6,7,8,9,10].

To examine memory, the Rey–Kim Memory Test, which is a complex method using verbal and visual tests, was employed. The verbal and figure aspects of memory were each tested and combined as the MQ (Figure 1). The FPEH treatment groups showed statistically significant dose-dependent increases in the MQ, an indicator of memory improvement. 

The learning gradient measures how verbal information is remembered in the KAVLT test. A higher percentage indicates a greater ability to remember. When compared to the placebo group, as the intake of FPEH increased, the learning gradient increased, but the increases were not statistically significant (Figure 2). However, the absolute number of words remembered during the first and last trials increased as the daily dose increased, indicating that FPEH was effective in improving memory input through repetition (Figure 3). A previous study in high-school-age children showed a significant increase in the learning gradient following the consumption of 200 mg of FPEH twice daily for 3 weeks [5]. The reason for the difference in results may be related to the differences in the ages of the two study populations, with an older population being involved in the current study. Differences in uptake, metabolism and peptide functionality may change during aging, as one possibility to help explain the differences between studies of younger and older subjects.

Memory retention indicates how well an individual maintains what was once remembered (Figure 4). The results of this study support the results of a previous study in healthy adults that demonstrated an increase in memory retention at doses of 200 and 400 mg of FPEH per day for 3 weeks [10]. In the current study, 400 and 600 mg of FPEH exhibited an increase in the total number of words remembered after 3 weeks.

Memory retrieval efficiency evaluates how well the study subject can utilize what is remembered (Figure 5). The results of the current study showed that the retrieval efficiency increased non-statistically as the daily intake of FPEH increased, with a maximum at a dose of 400 mg/day. A previous study reported small but significant increases in memory retrieval efficacy at doses of 200 and 400 mg/day [10]. The reason for the differences between the two studies is not known, in spite of the fact that a higher dose was used in the current study. The variance in results may relate to the differences in the ages of the study populations, with an older average population being used in the current study. 

Visual copying, drawing and recall ability are determined by the KCFT of the Rey–Osterrieth complex figure test. The KCFT is a widely used neuropsychological test that measures how well a complex figure is effectively remembered and drawn. Each subject is given the figure and is then asked to draw (copy) it. The figure is then removed and the subject is asked to draw the figure again (immediate recall). Twenty minutes later, the subject is asked to draw the figure from memory (delayed recall). A scoring system rates the accuracy, location and organization of each of the drawn figure components to the original, determining a composite score that estimates prefrontal lobe executive function from visuospatial perceptive/constructive ability and memory [18]. 

Scores can be affected by ages over 70 years and intelligence quotient (IQ) levels but not by gender or education [18,19,20,21,22]. In this study, six subjects (9.5%) were over age 70, and thus the KCFT test in this study was not overly influenced by age. The results of this study indicate that FPEH was effective in improving cognitive function for the integration of visuospatial memory and executive function (Figure 6). 

Overall, the results mirror and support the results of eight other studies [5,6,7,8,9,10]. Two of these trials involved school-age children that received 200 mg of FPEH twice daily for 4–16 weeks [7,8]. A randomized, controlled study involved healthy high school students given 200 mg of FPEH or a placebo twice daily for 3 weeks [5]. 

Three separate randomized, placebo-controlled studies with a combined total of 196 healthy adults (average age of about 42 years) reported that the administration of FPEH at doses of 200 mg either once or twice a day as compared to a placebo for 3 weeks resulted in dose-dependent increases in mental function measured by the Korean version of the Wechsler Adult Intelligence Scale (K-WAIS) and Rey–Kim Memory tests (MQ) [10]. Doses of FPEH as high as 600 mg were not used, and the average age was younger than in the current study. A randomized, controlled study of 25 seniors over 60 years of age in a day care center ingesting a placebo or 200 mg of FPEH twice daily for 3 weeks showed that Mini Mental State Examination, Korean version (MMSE-K) scores were significantly improved in the FPEH group as compared to the placebo group [6]. The results of the current study demonstrate the beneficial effects in a dose-dependent manner over a wider dosage range, and over a very wide age range, in healthy subjects, extending knowledge with respect to product efficacy as well as safety.

Many nutrients have been examined for their effects on mental function, particularly for delaying cognitive decline or reducing the risk of dementia. However, recent reviews have reported mixed effects on memory and/or cognition, ranging from null to moderate for antioxidants (beta-carotene, ascorbate and tocopherols), B vitamins, *Centella asiatica* extracts, cytidine diphosphocholine, docosahexaenoic acid (DHA) and omega-3 fatty acids, ginseng (*Panax ginseng* and other related plants) extracts, *Ginkgo biloba* extracts, multi-ingredient supplements, multiple vitamin–mineral combinations (MVMs), soy isoflavones, and vitamin D with calcium [23,24,25,26,27,28,29,30,31,32]. 

Recent reviews of pharmacological agents did not find improvements or a lessening of decline in the mental function of normal persons or those with mild cognitive impairment, but they did find an increased incidence of serious side effects [25,28]. Other nutrients such as alpha glycerophosphocholine [33], aquaeporin [34], *Bacopa monnieri* extracts [35,36,37,38], and DHA-rich phosphatidyl serine [39] have shown benefits for mental function in normal persons.

Comparison studies in humans on the effects of FPEH relative to other nutrients would provide perspective on the potential utility of FPEH. Despite heterogeneity in the study design, the type of subjects, mental status, the form of the material used, dosages, duration, and measurement tools, it is possible to derive comparative insights between FPEH and other nutrients. In general, FPEH has reproducibly produced significant improvements from baseline and from placebo groups over 3 weeks at doses of 200–600 mg daily in normal persons over a wide range of ages. Other nutrients have usually employed only aged subjects, subjects with dementia or Alzheimer’s disease, higher doses, longer times before effects are found, smaller quantitative improvements, fewer studies or inconsistent results between studies than FPEH. Thus, FPEH is a promising nutrient for relatively short-term benefits for mental function in a wide age range of normal, healthy subjects.

Several lines of evidence exist regarding mechanisms by which FPEH enhances memory and learning. In four healthy volunteers with an average age of 23 years, an open-label imaging study of brain blood flow (Single Photon Emission Computed Tomography (SPECT) scans) with the K-WAIS IQ test showed that 30 min after the ingestion of FPEH, brain areas that participate in learning and memory showed increased circulation and glucose uptake that correlated with increased IQ test scores [9]. In an in vitro system, FPEH protected human neuroblastoma (SK-N-SH) cells from reactive oxygen species [10,40]. The beta-sheet portions of FPEH may bind to beta-sheet portions of other proteins such as amyloid beta, preventing aggregation that is known to impair the brain functions of memory and learning [5,41,42,43,44,45,46]. When FPEH was fed or injected intraperitoneally to animals also injected intrathecally with beta amyloid, brain fibril and plaque formation was reduced [8]. Small peptides 5–11 amino acids long (the same range as found in FPEH) are called beta-sheet breakers [45,46,47], and they bind to the beta-sheet areas of beta-amyloid, blocking the ability to start the fibril/plaque formation cascade [40,41,42]. One retro-inverso peptide to the KLVFF amino acid sequence in beta-amyloid, made from D-amino acids and attached to a retro HIV protein transduction domain (TAT) to form RI-OR2-TAT peptide, was injected into mice peripherally and was shown to cross the blood–brain barrier and reduce beta-amyloid aggregation and its downstream effects [47]. Thus, beta-amyloid binding peptides are being studied for the prevention of the initial deleterious aggregation of beta-amyloid in vivo. The report by Lee et al. [9] illustrated improved circulation in human brain areas after the administration of FPEH. While this finding does not prove the passage through the blood–brain barrier, it does demonstrate that FPEH has the potential to interact with beta-amyloid and prevent sequelae that affect mental functions as one mechanism of action.

Another mechanism involves improving local brain circulation and glucose delivery, as suggested by the brain imaging study [9]. Preclinical studies in animal models have shown reductions in blood pressure from various hypertensive animal models [48], as well as improvements in glucose control and insulin release in vitro and in animal models [49,50]. These combined results suggest that FPEH, similarly to other peptides, may exert an effect on local microcirculation in active brain areas. A better blood supply in the brain could explain the preclinical and human clinical study results.

In summary, the results of this study indicate that the FPEH is effective in improving memory for verbal and visual tests in a dose-dependent manner. An intake of 400–600 mg was shown to improve memory. The results were observed in a study population between the ages of 28 and 92 years with an average age of about 55 years. The wide age range used in this study can be viewed as both an advantage and disadvantage. The results strongly support the observations of previous studies on FPEH, but they do so over a wider age range. The study also indicates for the first time that maximum beneficial effects are derived with a dose of 400–600 mg. The study also supports the safety of FPEH. 

Although positive and promising results were obtained with respect to the various aspects of memory enhancement, additional studies are required to provide clarity regarding the mechanism(s) of action and to determine whether biochemical biomarkers involved in memory and learning processes can be identified that correlate with the observed results. For example, plasma levels of brain-derived neurotrophic factor (BDNF), vascular endothelial growth factor (VEGF), insulin-like growth factor (IGF-1), cytokines and catecholamines can be assessed. In addition, studies in other ethnic groups are needed to affirm the effectiveness of FPEH in a broader spectrum of individuals, as all studies to date have been conducted in Korean populations.
